# Factors affecting beef quality and nutrigenomics of intramuscular adipose tissue deposition

**DOI:** 10.5713/ab.22.0380

**Published:** 2023-01-11

**Authors:** Myunggi Baik, Jaesung Lee, Sang Yeob Kim, Kamburawala Kankanamge Tharindu Namal Ranaweera

**Affiliations:** 1 Department of Agricultural Biotechnology and Research Institute of Agriculture and Life Sciences, Seoul National University, Seoul 08826, Korea; 2 Institute of Green-Bio Science and Technology, Seoul National University, Pyeongchang 25354, Korea

**Keywords:** Beef Cattle, Beef Quality, Genome-based Precision Feeding, Intramuscular Adipose Tissue Deposition, Marbling, Nutrigenomics

## Abstract

Beef quality is characterized by marbling (marbling degree and marbling fineness), physiochemical (shear force, meat color, fat color, texture, and maturity), and sensory (tenderness, flavor, juiciness, taste, odor, and appearance) traits. This paper summarizes and addresses beef-quality characteristics and the beef-grading systems in Korea, Japan, the USA, and Australia. This paper summarizes recent research progresses on the genetic and nutritional factors that affect beef quality. Intramuscular (i.m.) adipose tissue deposition or marbling is a major determinant of beef quality. This paper addresses the mechanisms of i.m. adipose tissue deposition focused on adipogenesis and lipogenesis. We also address selected signaling pathways associated with i.m. adipose tissue deposition. Nutrients contribute to the cellular response and phenotypes through gene expression and metabolism. This paper addresses control of gene expression through several nutrients (carbohydrates, fat/fatty acids, vitamins, etc.) for i.m. adipose tissue deposition. Several transcription factors responsible for gene expression via nutrients are addressed. We introduce the concept of genome-based precision feeding in Korean cattle.

## INTRODUCTION

Beef-quality and quantity characteristics are important, as they determine the price of beef and consumer preference. Beef quality is categorized by marbling, physiochemical, and sensory traits. Several factors affect beef quality aspects. These include genetic, management, and nutritional factors [1,2]. Carcass classification and grading systems are needed to standardize carcass characteristics and facilitate trading [3]. Several countries including Korea, Japan, USA, and Australia have established beef classification and grading systems [3].

Intramuscular (i.m.) adipose tissue deposition or marbling in the *longissimus dorsi* muscle (LM) is the most important trait affecting beef quality and palatability, as well as beef price, particularly in Korean cattle (Hanwoo) and Japanese Black cattle [3]. To enhance beef quality, understanding the molecular mechanisms of i.m. adipose tissue deposition is important. Functional genomics tools, including transcriptomics, proteomics, and nutrigenomics, have been used to elucidate the molecular mechanisms of i.m. adipose tissue deposition in beef cattle [2,4]. In addition, nutrigenomics is the combined study of genomics and nutrition [4]. Nutrigenomics may apply for maximizing the expression of the genetic potential of beef cattle.

In this paper, we review beef-quality characteristics, global beef-grading systems, factors that affect beef quality, and the molecular mechanisms responsible for bovine i.m. adipose tissue deposition. We also address the nutrigenomics aspects of i.m. adipose tissue deposition in cattle.

## FACTORS AFFECTING BEEF QUALITY

### Beef quality and yield characteristics

Beef-quality characteristics are categorized into nutritional, physiochemical, and sensory traits (Table 1). Beef-quantity characteristics include carcass weight, eye-muscle area, and backfat thickness.

Beef consists of water (70% to 76%), protein (15% to 23%), muscle/fat (0.7% to 38%), carbohydrates (0.5% to 2%), minerals (0.5% to 2%), vitamins, etc. [1,2]. The nutritional traits of beef include protein, fat, fatty acids, vitamins (e.g., B_3_, B_6_, and B_12_), iron, zinc, and selenium. Beef is an important source of amino acids in the human diet and provides a balanced amino-acid profile [5]. Beef is also a good source of amino acids with antioxidant properties and peptides for human health [5]. Beef contains extremely variable amounts of fat, ranging from 1.9% to 37.8%, depending on the breed (Brahman or Wagyu) and other factors [2].

The major categories of fatty acids in beef LM are saturated fatty acids (SFAs) and monounsaturated fatty acids (MUFAs) [6]. The major SFAs in the LM of Korean cattle steers are palmitic acid (C16:0; 25.8% to 27.4% of fresh loin fat) and stearic acid (C18:0; 9.7% to 10.4% of fresh loin fat) [6]. The most abundant MUFA in beef LM is oleic acid (C18:1; 43.1% to 46.6% of fresh loin fat in Korean cattle steers). The major polyunsaturated fatty acids (PUFAs) in the LM of Korean cattle steers are linoleic acid (C18:2n6; 0.49% to 0.61% of fresh loin fat), gamma-linolenic acid (C18:3n6; 0.02% to 0.06%), eicosatrienoic acid (C20:3n6; 0.06% to 0.08%), and eicosapentaenoic acid (EPA: C20:5; 0.14% to 0.17%) [6]. PUFAs are classified into omega-3 (n-3) fatty acids (double bond between the third and fourth carbons from the end methyl group) and omega-6 (n-6) fatty acids (double bond between the sixth and seventh carbons from the end methyl group [7]. The major omega-3 PUFA is alpha-linolenic acid, which is metabolized to EPA, docosapentaenoic acid (DPA, C22:5), and docosahexaenoic acid (DHA, C22:6). The omega-3 PUFAs may reduce the incidence of cardiovascular disease [8]. Linoleic acid is an abundant omega-6 that is metabolized to arachidonic acid (20:4n6) [8]. Pasture or organically raised beef contains higher percentages of total omega-3 fatty acids and PUFAs, which may be beneficial to human health [9].

Beef-quality grade (QG) affects the chemical composition of the LM. In the LM of Korean cattle, the protein percentage decreases with increasing QG, whereas the fat percentage increases [10]. With increasing QG, the percentages of loin MUFAs, including oleic acid, increase, whereas the percentages of PUFAs decrease [6,11]. Meanwhile, the SFA and MUFA composition of adipose tissue was regulated by adipose tissue fatty acid desaturation, with little contribution from hepatic or duodenal fatty acids [12]. Beef-cattle farmers are profoundly interested in carcass price, as it determines their income. In South Korea, the beef-carcass auction price is routinely determined by the wholesaler after beef grading in the slaughterhouse. In Korean cattle steers, the auction price is very strongly positively correlated with the marbling score (MS) ([R^2^ (regression coefficient of determination) = 0.75; p<0.001] and QG [R^2^ = 0.79; p<0.001]), confirming that MS or QG is a major determinant of beef price [13].

**Figure 1:**
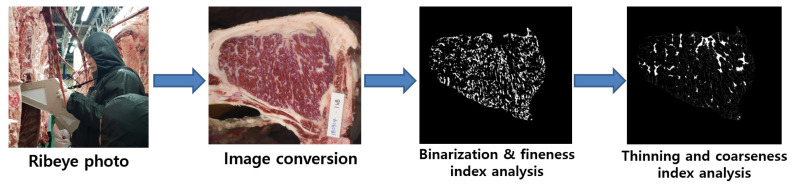
Computer imaging analysis procedure for the beef-marbling particles (MPs). Ribeye photograph was taken with an HK-333 camera, and the image was converted by binarization and thinning using Beef Analyzer II software (Kuchida et al [98]; Beak et al [13]). The MPs were categorized into fine MPs (0.01 to 0.5 cm^2^) and coarse MPs (>0.5 cm^2^).

Marbling is characterized by the presence of white particles or flecks of i.m. adipose tissue between the LM muscle bundles [14]. The size (fine or coarse) and spatial distribution of the marbling particles (MPs) are important marbling traits, in addition to the degree of marbling [15,16]. Consumers desire finely marbled beef, which improves the sale of Korean cattle [16,17]. Finely marbled beef is also favored by Japanese consumers and carries a higher price than coarsely marbled-beef [18]. Computer image analysis has been applied to measure the size characteristics (fineness or coarseness) of the MPs as well as the degree of marbling in the muscle (Figure 1) [15]. We recently evaluated the size characteristics of beef MP using computer image analysis in Korean cattle. The results showed that the size (fine or coarse) of the MPs was strongly positively correlated with auction price [13]. These marbling-trait characteristics were strongly associated with the auction price in highly marbled beef (QGs 1+ and 1++) but not in moderately marbled beef (QG 1). This study demonstrates that computer image analysis is a valuable tool for assessing the size characteristics of marbling traits and that it can be used to determine finely and highly marbled beef. We compared the transcriptomes of high- and low-marbling-fineness groups of *longissimus thoracis* (LT) muscle from Korean cattle. We identified 328 differentially expressed genes, analysis of which revealed that pathways regulating adipocyte hyperplasia and hypertrophy are involved in the marbling fineness of the LT [19].

The color of meat is evaluated firstly by consumers; thus, it is an important parameter influencing the purchasing decision [20]. At the point of sale, consumers generally cannot evaluate meat quality without opening the package. Thus, a bright cherry-red color is normally used as an indicator of the wholesomeness of fresh meat [21]. Any deviation from the bright cherry-red color of fresh meat can result in less consumer acceptance [22]. Myoglobin is the sarcoplasmic heme protein that is primarily responsible for meat color, but hemoglobin and cytochromes also contribute to a lesser extent [22]. Meat color is routinely measured using the Commission Internationale de l’Eclairage L*a*b* system [23], in which L*, a*, and b* represent the lightness, redness, and yellowness of the meat, respectively. Beef color is affected by several factors, including genetics, animal age, nutritional status, the slaughter process, and meat aging [1]. Fresh beef normally has a bright red color, which darkens with age, changing from bright red to dark red [24]. Diet can affect beef or carcass color. For example, the subcutaneous adipose tissue of steers fed grass is more yellow than that of animals fed concentrates [25]. This difference may be due to there being a greater amount of beta-carotene in pasture than in concentrates. Muscle from grain-finished Brahman steers is less dark and is redder in color than that from pastured steers [26]. Dietary vitamin E supplementation has been used to enhance the lipid and color stability of beef, and the antioxidant function of alpha-tocopherol may, in part, be responsible for this beneficial effect [27].

Beef eating quality is measured by sensory testing, and the sensory evaluation is performed by either trained panelists or untrained consumers. Sensory traits include tenderness, flavor, juiciness, taste, odor, appearance, and overall acceptance. Sensory traits are important determinants of the acceptability and palatability of beef, and a lexicon describing the flavor characteristics of beef was developed [28]. Tenderness is regarded as the most important consumer-satisfaction factor, followed by flavor and juiciness [29]. Meat tenderness depends on several factors, including background toughness related to connective tissue, the degree of muscle contraction, muscle myofibril degradation by proteolysis during aging, i.m. adipose tissue content, and protein denaturation during cooking [30–32]. The Warner-Bratzler shear force is an instrumentally measured value of tenderness. Meat flavor is a complex sensation that involves a combination of olfactory, gustatory, and trigeminal sensations that detect basic taste and aroma [33]. Meat juiciness is characterized by the perceived amount of juice and the level of lubrication during mastication of the meat in the mouth [30].

Beef yield is determined by several criteria, depending on the country [30]. In Korea and Japan, yield grade (YG) is characterized by the edible percentage of the meat, and is determined by the combination of carcass weight, eye-muscle area, and backfat thickness (or subcutaneous adipose tissue thickness) [3]. The basis of yield grading in the United States Department of Agriculture (USDA) system is an indication of the yield of boneless, trimmed retail cuts [3].

### Global beef-grading systems

Beef-grading systems are well established in several countries, including Korea, Japan, the USA, Australia, and the EU (Table 2) [3]. QG and YG are determined by carcass-based grading systems in Korea, Japan, and the USA. Meat Standards Australia (MSA) grading standards are based on cuts [3].

In Korea, beef is evaluated using two grading categories (QG and YG) based on the Korean Beef Carcass Grading System developed by the Korea Institute for Animal Products Quality Evaluation (KAPE) (Table 2) [34]. There are five levels of QGs: 1++ (best), 1+, 1, 2, and 3 (worst) [34]. The major QG item is MS or i.m. adipose tissue content, and other factors (meat color, fat color, and maturity) also affect the QG. The MS is scored on a nine-point scale, from 1 (devoid) to 9 (abundant). The best QG1++ includes MS 7, 8, or 9, whereas the worst QG3 has MS 1. Further, meat color, fat color, texture, and maturity also affect on QG. The three YG levels are A (highest), B, and C (lowest). The YG is determined by a combination of rib-eye area, backfat thickness, and carcass weight. In the YG system, six different yield-index equations were based on combination of three sex categories (cow, bull, and steer) and two breeds (Korean cattle and Holstein). Quality grades significantly affect carcass characteristics and fatty acids in Korean cattle steer loin [6,10]. For example, the i.m. fat% increases but protein% decreases as QG increases in the loin of Korean cattle steers. With increasing QG, oleic acid content (g/kg fresh meat) increases but the PUFA/SFA ratio decreases.

Japan uses the Japanese Meat Grading Association (JMGA) grading system, in which quality is graded based on marbling, meat color, meat brightness, meat texture, fat firmness, fat texture, fat color, fat luster, and fat quality [18]. In the JMGA system, beef QG is assessed after quartering between the fifth and sixth ribs, and there are 5 QG levels (5 [highest] to 1 [lowest]) [35]. There are 12 marbling levels (1 to 12; larger more abundant) and 3 YG levels (A [best], B, C [worst]) in the JMGA system, in which YGs are determined by a regression equation using the combined inputs of carcass weight, eye-muscle area, rib thickness, and fat thickness [35].

The USA uses the USDA grading system, in which eight beef QGs are currently applicable to steer and heifer carcasses: Prime, Choice, Select, Standard, Commercial, Utility, Cutter and Canner [3,36]. The USDA grading system has five YGs (1 to 5). Quality is graded based on marbling, ossification score, meat color, and meat texture. The USDA grading system has nine marbling levels from 1 (devoid) to 9 (abundant) [3]. Higher grading levels are characterized by higher marbling levels at lower maturity. For example, Prime carcasses need a minimum of slightly abundant marbling for A maturity, whereas Choice carcasses can have a minimum of small marbling for A maturity but need a minimum of modest marbling for B maturity [36]. The USDA YGs have five levels (1 to 5), and these are based on a regression equation with the combined inputs of the external fat amount; the amounts of kidney, pelvic, and heart fat; the quartered LM area; and the hot carcass weight [36].

The MSA system has unique features: it is a cut-based grading system defined by consumer-score outcomes and an eating-quality program, and is not separated by QG and YG [37]. The MSA has three grading levels of good for every day (three stars), better than for every day (four stars), and premium quality (five stars). Grading is based on several items, including USDA marbling, carcass weight, rib-fat depth, ossification score, meat color, *bos indicus* %, sex, hormonal-growth-promoter implants, milk-fed vealer, sale yard, hump height, hang technique, electrical stimulation, ultimate pH, days aged, cut type, and cooking method [37].

### Factors affecting beef quality

Several factor such as genetic, management, and nutritional factors affect the meat quality of cattle and buffalo (Table 3) [1,2,38,39]. The genetic factors include breed and sex. A comparison of i.m. fat contents among several breeds found that Japanese Wagyu beef had the highest i.m. fat content (36.05%), and Korean cattle (known as Hanwoo) beef had the second highest (14.1%), whereas Brahman beef had the lowest i.m. fat content (2.8%) in the LM [2]. Wagyu beef having the highest i.m. fat content among four cattle breeds (Wagyu, Angus, Brahman, and the Malaysian local breed Kedah-Kelantan) was confirmed in another study [40]. Beef price is largely dependent on the degree of marbling. For example, in 2022, Japanese Wagyu beef was the most expensive (18.30 USD/kg) in the world, and Hanwoo beef was the second-highest priced (16.50 USD), followed by American beef (9.30 US dollars), and Australian beef (7.20 USD) (Japan, [41]; Korea, [34]; USA, [42]; Australia, [43]). The beef price of Korean cattle varies widely depending on the QG. For example, the 2021 wholesale market beef price of QG 1++ beef was 19.50 USD/kg, whereas the price of QG 3 beef was 10.10 USD/kg, or almost half the price of QG 1++.

We compared several beef-quality traits among Korean cattle, Angus, and Holstein steers. The Korean cattle LT had the highest fat content; the highest percentage of MUFAs, including oleic acid; the lowest shear force; and the best sensory traits (flavor, tenderness, juiciness, and overall acceptance) among the three cattle breeds [44]. The i.m. adipose tissue content positively affects the sensory quality [10,45]. In addition to the i.m. adipose tissue content, the composition and content of fatty acids are important factors in beef palatability [46]. Oleic acid may be positively associated with beef flavor, whereas PUFAs may be negatively associated with beef flavor [47]. Thus, the relatively high i.m. fat and oleic-acid contents observed in our study may positively affect the sensory traits of Korean cattle beef [44]. Grain-fed beef had higher percentages of oleic acid than grass-fed beef [48].

Management factors, such as castration and the environment (e.g., temperature and season) affect beef quality and quantity. Castration profoundly affects beef quality, as it improves QG [49]. The majority of beef from uncastrated bulls is QG 3 (lowest QG), whereas over 80% of castrated steer beef from Korean cattle is equal to or above QG 1. Marbling scores are markedly higher in Korean cattle steers (MS = 1.1) than in bulls (MS = 5.0). Heifers and steers have higher carcass-fat contents compared to bulls in crossbred Holstein-Friesian ×Limousin cattle [50]. The castration method (half-castration or complete castration) affects beef quality and quantity. Half castration produces higher meat yields compared to complete castration (steers); it also produces meat with a higher IMF content and a lower shear force (higher tenderness) than that of uncastrated Korean cattle bulls [51].

Environmental factors, such as temperature and the season, can affect beef-quality characteristics. Piao and Baik [52] evaluated whether climatic conditions affected the beef-carcass characteristics of Korean cattle steers. Among the four seasons, backfat thickness was greatest in winter (December, January, February) and the grade-A yield percentage was lowest, whereas the YG C percentage was highest, indicating that YG is worse in winter. This is likely due to backfat thickness being highest in winter. Strategies that minimize the adverse effects of cold stress on YG are needed. We have investigated the effects of temperature on the growth of Korean cattle steers [53]. Mild or moderate cold stress did not affect the growth performance of Korean cattle steers at the early fattening stage. Cold temperatures increased concentrate and forage intakes/kg of body weight in our study. Feed intake also increases in cattle during the colder months [54]. Cold stress reduces growth performance and feed efficiency due to the increased energy required to maintain body temperature [55]. Increased feed intake may have contributed to maintaining body temperature during the cold conditions and provided the additional nutrients needed by the animals during the cold period, resulting in no changes in weight gain or feed efficiency in our study. A long photoperiod improves fat deposition by regulating the expression of lipid-metabolism-related genes in Jinjiang cattle during winter [56].

Nutritional factors affect beef quality. Lipogenesis contributes to i.m. adipose tissue content, affecting beef quality. Lipogenesis (*de novo* fatty acid synthesis) in ruminants generally occurs in adipose tissue via conversion of acetate and glucose to fatty acids [57,58]. Acetate is abundantly produced during ruminal fermentation and is used as a substrate for lipogenesis. In ruminants, glucose can be derived from either gluconeogenesis from propionate/lactate or from glucose absorbed by the small intestine [58]. ATP citrate lyase is involved in cleaving citrate to oxaloacetate and acetyl-CoA and is a key enzyme that is responsible for utilizing glucose during lipogenesis. The activity of ATP citrate lyase is low in ruminants, so glucose is less often used as a lipogenic substrate in ruminants than in monogastric animals [59]. It has been suggested that glucose is preferred to acetate as a substrate of lipogenesis for i.m. adipose tissue deposition [57], and sufficient activities of ATP citrate lyase were detected in bovine adipose tissue [60]. However, in a study of Angus × Simmental steers, acetate was more effective than glucose as the substrate for lipogenesis in intramuscular, subcutaneous, and visceral adipose tissue depots [61]. Beet pulp, a byproduct of sugar-beet processing that contains up to 40% neutral detergent fiber and approximately 23% pectin, produces more acetate and less propionate than corn grain [62,63]. We investigated the effects on lipogenic parameters of partially substituting beet pulp for corn grain in Korean cattle steers [64] and found that such substitution increased the proportion of ruminal acetate and circulating insulin levels, indicating that feeding beet pulp increased the lipogenic parameters. Triglycerol synthesis requires a glycerol backbone, which is primarily supplied by glucose [65]. Providing propionate for glucose synthesis via gluconeogenesis may also contribute to triglycerol synthesis. Therefore, large proportions of concentrates or grain are routinely included in the diet when fattening beef cattle [66–69]. Supplementing the finishing diet with 100 ppm γ-aminobutyric acid was found to improve antioxidant enzyme status in the *longissimus lumborum* of Korean cattle steers [70].

## MECHANISMS OF INTRAMUSCULAR ADIPOSE TISSUE DEPOSITION

The ratio of muscle to adipocytes in the LM determines the amount of i.m. adipose tissue deposition. Thus, understanding the mechanisms responsible for initial muscle- and adipocyte growth during the developmental stage of cattle is important. Adipogenesis includes the commitment of mesenchymal stem cells to preadipocytes, determination and proliferation of preadipocytes, and differentiation of preadipocytes into mature adipocytes [71]. Both hyperplasia (increased cell numbers) and hypertrophy (increased cell size) are associated with i.m. adipose tissue deposition [72]. May et al [73] provided direct evidence for intramuscular adipose hyperplasia in Angus and Wagyu steers. Hyperplasia of adipocytes occurs from 12 to 16 months of age in Angus steers [74], or from 13 to 18 months of age in beef cattle [75]. It has also reported that adipocyte hyperplasia continued throughout the life of beef cattle [76]. The visible marbling is formed by the combined processes of hyperplasia and hypertrophy [75,76]. Strategies to increase hyperplasia and hypertrophy of adipocytes may help to improve i.m. adipose tissue deposition [2]. Harper and Pethick [77] suggested that selecting cattle with high genetic potential to produce more preadipocytes within muscle is important for increasing i.m. adipose tissue deposition.

Our laboratory focused on understanding the molecular mechanisms of i.m. adipose tissue deposition. We mainly used the castration model, as castration significantly increased i.m. adipose tissue deposition in Korean cattle [2,49]. Quantitative real-time polymerase chain reaction analysis revealed that steers had higher LM mRNA levels of genes involved in lipid uptake (lipoprotein lipase, *CD36*) and lipogenesis [acetyl-CoA carboxylase (*ACC*), fatty acid synthase] compared with bulls [49], whereas steers had lower LM mRNA levels of genes involved in lipolysis (adipose triglyceride lipase, monoglyceride lipase), although hormone-sensitive lipase was higher in steers than in bulls [49]. Furthermore, we found that LM expression levels of some fatty-acid-uptake, lipogenesis, and fatty-acid-esterification genes were positively correlated with i.m. fat content, whereas the LM expression levels of some lipolysis genes were negatively correlated with i.m. fat content [78].

We also used microarray analysis to examine transcriptomic changes in the LM following castration of Korean cattle bulls. Castration upregulated the transcriptomes involved in lipid metabolism, including adipogenesis, fatty-acid-synthesis/-esterification and fatty-acid-oxidation, tricarboxylic-acid-cycle, and oxidative-phosphorylation genes [79]. We also applied RNA-sequencing analysis to examine transcriptomic changes in Korean cattle bulls following castration and found that the transcriptomes involved in known pathways such as peroxisome proliferator-activated receptor signaling and retinol metabolism changed in the LM [80]. We also found that the transcriptomes of novel pathways such as the complement and coagulation cascades are changed. Our study demonstrated that the complement and coagulation cascade pathways may be involved in IMF deposition.

We compared the expression of genes involved in extracellular matrix (ECM) and integrin genes in the LT between Korean cattle bulls and steers. Steers had lower collagen type 1 α1 and collagen type 3 α1 mRNA levels than bulls, but they had higher matrix metalloproteinase 9 (MMP9) mRNA levels [81]. Steers had higher integrin α5 mRNA levels, but lower integrin β6 mRNA and protein levels. Regression analysis showed that MMP9 mRNA levels were positively correlated with IMF content. Our findings implied that some ECM-related factors may be involved in IMF deposition. In another study, myosin heavy chain isoforms partially accounted for the variations in meat quality between different Thai native cattle breeds [82]. Overall, these findings imply that the combined effects of increased lipogenesis, increased fatty-acid uptake, increased fatty-acid esterification, decreased lipolysis, and changes in ECM-related gene expression contribute to increasing IMF deposition. Therefore, designing methods (nutritional, managerial, or genetic) that increase fat deposition but decrease fat removal may be effective for enhancing IMF deposition in the LM.

We have also examined whether castration affects adipose cellularity and lipid-metabolism gene expression in various fat depots. Castration increased body-fat cell sizes in various fat depots, including subcutaneous, abdominal, and perirenal fat. Upregulation of adipogenesis (CCAAT/enhancer binding protein alpha, ACC) and down-regulation of fatty-acid β-oxidation (medium-chain acyl-CoA dehydrogenase) genes may partially contribute to increased adiposity [83]. We also compared hepatic expression levels of lipid-metabolism genes between Korean cattle bulls and steers. Steers had higher hepatic ACC and sterol regulatory element binding protein 1 mRNA levels than bulls [84]. However, castration did not significantly affect the hepatic gene expression involved in TG synthesis, fatty-acid oxidation, and very-low-density lipoprotein secretion. Overall, our studies demonstrate that lipid metabolism in the LM is important for the regulation of IMF deposition, whereas hepatic lipid metabolism has minor effects on IMF deposition.

**Figure 2:**
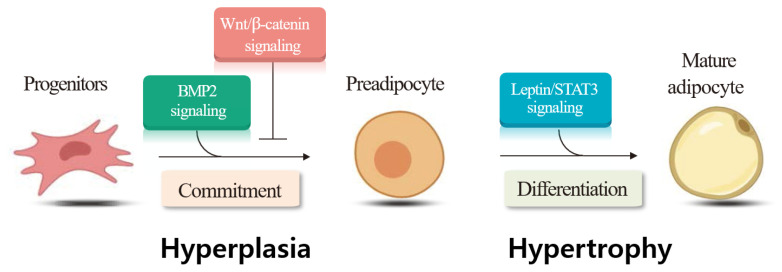
Changes in the hyperplasia and hypertrophy signaling pathways in the *longissimus dorsi* muscle of Korean cattle bulls after castration (Jeong et al [85]; Jung and Baik [86]). Wnt, Wingless and Int; BMP2, bone morphogenetic protein 2; STAT3, signal transducer and activator of transcription 3.

We examined the gene expression involved in several signaling pathways following castration of Korean cattle bulls. We found decreases in Wingless and Int (Wnt)/beta-catenin signaling pathway genes (wingless-type MMTV integration site family, member 10b; cadherin-associated protein, beta 1), but increases in Wnt antagonist (secreted frizzled-related proteins 4) and adipogenic (peroxisome proliferator-activated receptor gamma) gene expression following castration [85]. Our findings imply that downregulation of the Wnt/beta-catenin signaling pathway following castration may upregulate adipogenic gene expression, thereby contributing to i.m. adipose tissue deposition in the LM. We also evaluated whether castration affected bone morphogenetic protein 2 (BMP2) levels and the expression of its signaling molecules in Korean cattle, and detected higher plasma BMP2 and leptin levels in steers than in bulls [86]. In the same study, steers had higher mRNA levels of the lysyl oxidase gene, a downstream target of the BMP signaling pathway, adipogenic peroxisome proliferator-activated receptor gamma, and lipogenic fatty acid binding protein 4 genes in the LT, compared to bulls. The study demonstrated that upregulation of the BMP signaling pathway in response to castration may increase adipogenic gene expression, contributing to i.m. adipose tissue deposition in castrated animals. Overall, our findings demonstrate that the expression of genes involved in signaling pathways for hyperplasia (Wnt/beta-catenin, BMP2 signaling) and hypertrophy (leptin/signal transducer and activator of transcription 3 signaling) changed in the LM following castration (Figure 2).

## NUTRIGENOMICS OF INTRAMUSCULAR ADIPOSE TISSUE DEPOSITION

**Figure 3:**
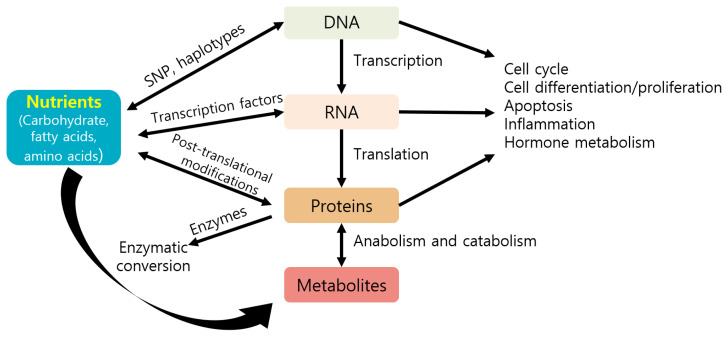
Contribution of nutrients to the cellular response and phenotypes by regulating several gene expression steps (modified from Costa et al [87]). SNP, single nucleotide polymorphism.

**Figure 4:**
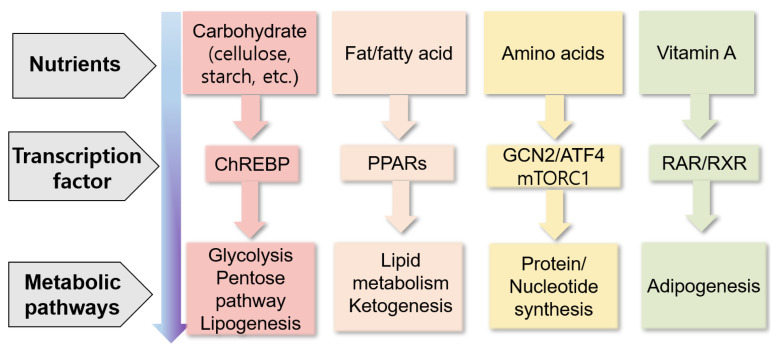
Summary of the control of gene expression through nutrients via transcription factors and their metabolic responses (modified from Haro et al [88]). ATF4, activating transcription factor 4; ChREBP, carbohydrate-responsive element binding protein; GCN2, general control non-derepressible 2; mTORC1, mechanistic target of rapamycin complex 1; PPARs, peroxisome proliferator-activated receptors; RAR, retinoic acid receptor; RXR, retinoic X receptor.

Nutrients can directly or indirectly regulate gene expression at several steps, including transcription, translation, and post-translational modifications, thereby affecting several cellular responses (e.g., cell cycle, inflammation, and metabolism) and phenotypes of animals (Figure 3) [87]. Nutrients may control gene expression via transcription factors, affecting nutrient metabolism (Figure 4) [88]. For example, carbohydrates may regulate gene expression via the transcription factor carbohydrate-responsive element binding protein, affecting metabolic pathways, such as lipogenesis. Lipids or fatty acids may control gene expression via the transcription factor peroxisome proliferator-activated receptor, affecting lipid metabolism. Amino acids may control gene expression via the general control nonderepressible 2/activating transcription factor 4 and mechanistic target of rapamycin c1 pathways, affecting protein synthesis. Vitamin A may regulate gene expression via the transcription factors retinoic acid receptor/retinoic X receptor heterodimer, affecting adipogenesis.

**Figure 5:**
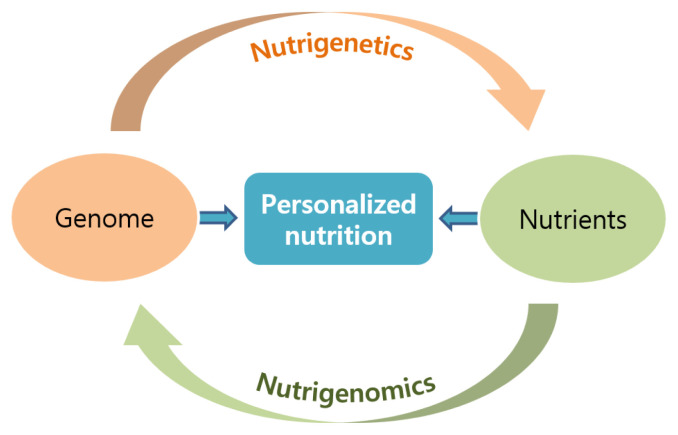
Interaction between nutrients and genes or the genome (modified from Mutch et al [89]). The genome affects how the body responds to nutrients, and nutrients affect gene expression. This interaction can be applied to design personalized diets for humans and animals.

Functional genomic tools including transcriptomics, proteomics, and metagenomics, and fusion technologies such as nutrigenomics have been applied in animal studies. Nutrigenomics and nutrigenetics represent the interaction between nutrients and genes/genome (Figure 5) [89]. The genome affects how the body responds to nutrients, and nutrients affect gene expression. This interaction can be applied to the design of personalized diets for humans and animals. Nutrigenomics is defined as the application of high-throughput genomic tools, such as transcriptomics, to nutrition research [90]. Nutrigenomic studies have been conducted on lipogenesis and beef quality. For example, pre- and post-natal nutritional modulation and weaning age resulted in transcriptomic changes associated with lipogenesis and inflammation in beef cattle [91]. Metabolomic and transcriptome analyses revealed that finishing with a forage diet affected the metabolic pathways related to tenderness and i.m. fat contents as compared to finishing with a grain diet in cattle [92]. Nutrigenetics is defined as analysis of the effects of genetic variations on the interaction between nutrients and diseases or phenotypes [93]. Nutrigenetic interactions between the alcohol dehydrogenase 1C (*ADH1C*) single nucleotide polymorphism (*ADH1C* c.-64T>C) and vitamin A levels have been studied. ADH is an enzyme that oxidizes retinol to retinaldehyde, and retinaldehyde inhibits adipocyte differentiation [94]. Cattle with the TT genotype had 22.9% greater i.m. fat contents compared with those with the CC genotype without vitamin A supplementation [95].

**Figure 6:**
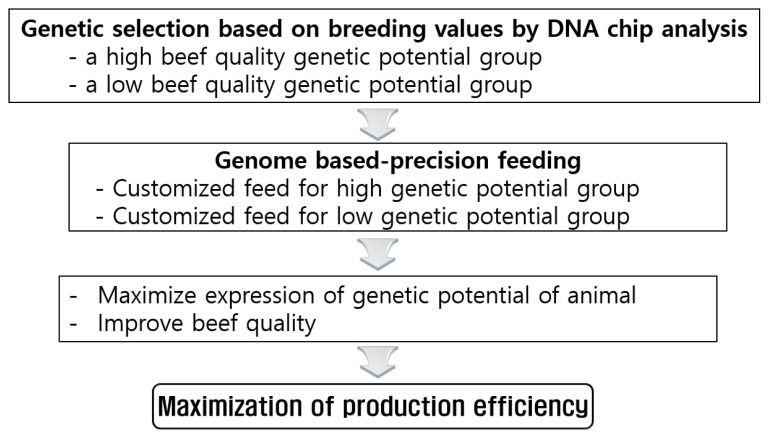
Nutrigenetic study on beef quality: genome-based precision-feeding model of Korean cattle.

Previously, we have proposed nutrigenetic study concept of Korean cattle: “Genome-based precision feeding model” [4]. The study may be feasible because Korean cattle are genetically heterogenous, so that genetic selection is still possible. Selection of high- or low- beef quality genetic potential groups may be made, based on breeding values by DNA chip analysis (Figure 6). Genome based-precision feeding may be made by customized feed for high vs low genetic potential group. This approach may maximize expression of genetic potential of animal and may improve beef quality, leading to maximization of production efficiency.

## CONCLUSION

Several factors affect beef quality, including genetics, management, and nutrition. These factors should be considered to improve beef quality, including marbling and tenderness. Other factors, such as greenhouse gas emissions/carbon footprint, animal welfare, safety (antibiotics), and rural development should also be considered in the future. Hyperplasia and hypertrophy of adipocytes may be involved in i.m. adipose tissue deposition. The combined effects of lipogenesis, fatty-acid uptake, fatty-acid esterification, and lipolysis contribute to i.m. adipose tissue deposition. The Wnt/beta-catenin, BMP2, and leptin/signal transducer and activator of transcription 3 signaling pathways may regulate i.m. adipose tissue deposition. These results will be useful in the design of efficient methods to improve beef quality. Nutrients regulate gene expression via transcription factors, affecting metabolism and the phenotype. Genome-based precision feeding may maximize the expression of the genetic potential of an animal and improve the production efficiency and beef quality of Korean cattle. The combined effects of the genome, the environment, the rumen microbiome, and the epigenome, and their interaction, determine the cattle phenotype. Thus, a systemic approach using multi-omics may provide an integrated solution to improve economic traits, including beef quality as well as growth.

## Figures and Tables

**Table 1:** Summary of beef quality and yield characteristics1)

Criteria	Items or Indicators
Beef quality characteristics	
Marbling	Marbling score, marbling size (fine, coarse)
Physiochemical traits	pH, cooking loss, shear force, meat color, fat color, texture, maturity
Sensory traits	Tenderness, flavor, juiciness, taste, odor, appearance, overall acceptance
Beef quantity characteristics	
Yield traits	Carcass weight, eye muscle area, backfat thickness
Other considerations	
Economic efficiency	Income
Social consideration	Rural development
Environmental consideration	Carbon footprint, animal welfare
Safety issue	Antibiotic

1) Modified from Hocquette et al [14].

**Table 2:** Summary of global beef grading systems of several countries1)

Country (Grading scheme: unit)	Quality grade			Yield grade	
	
	Grades	Basis of grading	Marbling level	Grades	Basis of grading
South Korea (Korea: carcass)	Location: 13th rib interface 5: 1++, 1+, 1, 2, 3	Marbling, meat color, fat color, texture, maturity (ossification score)	9: 1–9 (larger more abundant)	3: A, B, C	Carcass weight, eye muscle area, backfat thickness
Japan (JMGA: carcass)	Location: 6th-7th rib section 5: 5, 4, 3, 2, 1	Marbling, meat color, meat brightness, meat texture, fat firmness, fat texture, fat color, fat luster, fat quality	12: 1–12	3: A, B, C	Carcass weight, eye muscle area, rib thickness, fat thickness
USA (USDA: carcass)	8: Prime, Choice, Select, Standard, Commercial, Utility, Cutter, Canner	Marbling, ossification score, meat color, meat texture	9: Abundant, moderately abundant, slightly abundant, moderate, modest, small, slight, traces, practically devoid	5: 1, 2, 3, 4, 5	Carcass weight, eye muscle area, rib fat, kidney and perirenal fat
Australia2) (MSA: cut)	3: good everyday (3 star), better than everyday (4 star), or premium quality (5 star)	Bos indicus %, sex, HGP implants, milk fed vealer, sale yard, ccarcass weight, hump height, hang technique, electric stimulation, USAD marbling, rib fat depth, ossification score, meat color, ultimate pH, days aged, cut types, cooking method	USDA marbling score	-	-

1) Sex is considered for all grading systems.

2) MSA grading standards are defined by consumer score outcomes and eating-quality program, and not separated for quality and quantity grades.

JMGA, Japanese Meat Grading Association; MSA, Meat Standards Australia; USDA, United States Department of Agriculture; HGP, hormone growth promotants implant.

Source: Polkinghorne & Thompson [3]; Motoyama et al [18]; Bonny et al [37]; Jo et al [66]; Farmer & Farrell [96]; Joo et al [97].

**Table 3:** Summary of factors affecting beef quality

Factors	Major beef quality items
Genetic factors	
Breed	Marbling, sensory, yield, income
Sex	Marbling, sensory, musculature, income
Management factors	
Castration	Marbling, sensory, yield, income
Slaughter age/weight	Marbling, sensory, yield, income
Environments	Marbling, yield
Nutritional factors/feeding system	
Plane of nutrition	Marbling, sensory, yield, income
Roughage-concentrate ratio	Marbling, fatty acid composition, sensory, income
Feeding systems	Marbling, sensory, income

Source: Sakowski et al [1]; Park et al [2].
